# Neurocognitive Impairment in Idiopathic Pulmonary Fibrosis: A Systematic Review of Current Evidence

**DOI:** 10.3390/medsci13040288

**Published:** 2025-11-27

**Authors:** Dacian Mihart, Alexandru Florian Crisan, Vlad Carunta, Daniel Trăilă, Emanuela Tudorache, Cristian Oancea

**Affiliations:** 1 Doctoral School, “Victor Babes” University of Medicine and Pharmacy Timisoara, Eftimie Murgu Square 2, 300041 Timisoara, Romania; 2 Research Center for the Assessment of Human Motion, Functionality and Disability (CEMFD), “Victor Babes” University of Medicine and Pharmacy Timisoara, Eftimie Murgu Square 2, 300041 Timisoara, Romania; 3 Pulmonary Rehabilitation Center, Clinical Hospital of Infectious Diseases and Pulmonology, “Victor Babes”, Gheorghe Adam Street 13, 300310 Timisoara, Romania; 4 Center for Research and Innovation in Personalized Medicine of Respiratory Diseases (CRIPMRD), “Victor Babes” University of Medicine and Pharmacy Timisoara, Eftimie Murgu Square 2, 300041 Timisoara, Romania; 5 Pulmonology Clinic, Clinical Hospital of Infectious Diseases and Pulmonology, “Victor Babes” University of Medicine and Pharmacy Timisoara, Gheorghe Adam Street 13, 300310 Timisoara, Romania

**Keywords:** idiopathic pulmonary fibrosis, cognitive impairment, working memory, neuropsychological assessment, executive function

## Abstract

Background: Idiopathic pulmonary fibrosis (IPF) is a progressive disease with a major impact on respiratory function, but also with possible underestimated effects on cognitive function. Although interest in cognitive impairment in chronic respiratory diseases, such as COPD, has increased, data on IPF remain limited and heterogeneous. Objective: This systematic review aimed to synthesize current evidence on cognitive impairment in IPF, identify the most affected domains, and evaluate the certainty of evidence using standardized methodological tools. Methods: A systematic review was conducted according to PRISMA 2020, with a registered PROSPERO protocol (CRD420251041866). Four databases (PubMed, Scopus, Web of Science, Cochrane Library) were searched for studies from 2014 to 2025. Methodological quality and certainty of evidence were appraised with the Joanna Briggs Institute (JBI) and GRADE frameworks. Results: Four studies met the inclusion criteria (two cross-sectional, one descriptive, one case–control). Across investigations, working and verbal memory emerged as the most consistently impaired domains, followed by processing speed and executive function, whereas visuospatial and language abilities were less frequently affected. Cognitive impairment was present even in mild IPF and became more pronounced with lower DLCO, shorter 6 min walk distance, greater desaturation, and obstructive sleep apnea. Certainty of evidence ranged from low to moderate due to small samples and heterogeneous testing. Conclusions: Cognitive dysfunction, particularly in memory, attention, and executive domains, is a frequent but under-recognized feature of IPF. Routine screening with brief, validated tools such as the MoCA may facilitate early detection and guide individualized rehabilitation.

## 1. Introduction

Idiopathic pulmonary fibrosis is a severe and progressive form of interstitial lung disease (ILD), characterized by irreversible fibrous remodeling of the lung parenchyma, loss of alveolar elasticity, and impaired gas exchange [1]. The disease mainly affects older adults and progresses slowly but steadily to respiratory failure and death. Despite therapeutic advances in recent decades, the prognosis remains poor, with median survival estimated at 3–5 years from diagnosis [2]. Worldwide, the prevalence of idiopathic pulmonary fibrosis (IPF) ranges from 6.3 to 71 cases per 100,000 inhabitants, depending on the diagnostic criteria and the population studied [3].

Most patients with IPF are elderly and have multiple comorbidities, which complicates clinical management and negatively impacts quality of life. Common comorbidities include cardiovascular disease, depression, obstructive sleep apnea, and neurological disorders [4]. In particular, research interest in cognitive impairment in chronic respiratory diseases has increased in recent years, especially in chronic obstructive pulmonary disease (COPD), where significant cognitive impairment has been demonstrated even in patients without severe hypoxemia [5]. In contrast, the literature investigating cognition in IPF is extremely limited, and the mechanisms involved are poorly understood.

Existing evidence suggests that patients with IPF may present with a wide range of cognitive deficits, including impairments in memory, attention, language, psychomotor coordination, and executive functions [6,7]. A prospective study reported the presence of mild cognitive impairment in almost half of patients with IPF, in the absence of severe hypoxemia [8]. Also, the use of instruments such as MoCA or extended neuropsychological batteries revealed significantly lower scores in patients with IPF compared to controls [9].

The involvement of factors such as chronic hypoxemia, systemic inflammation, decreased alveolar–capillary diffusing capacity (DLco), and the presence of OSA suggests a multifactorial contribution to cognitive decline in IPF [10,11]. In addition, systemic factors such as frailty, sarcopenia, corticosteroid use, and physical inactivity may contribute to neurocognitive degradation, further affecting the patient’s daily functionality [10].

Cognitive impairment in IPF is not just an isolated comorbidity, but a problem with major clinical impact. Cognitive dysfunctions can limit the patient’s ability to understand medical indications, comply with treatments, actively participate in therapeutic decisions, or monitor symptoms and adverse reactions [7]. In addition, they can increase the risk of social isolation, depression, and repeated hospitalizations, affecting both the patient and his or her family [4]. However, in current clinical practice, cognitive screening is not systematically integrated into the assessment of patients with IPF.

Another essential problem is the lack of a standardized protocol for assessing cognition in IPF. The instruments used in existing studies vary considerably, from general questionnaires Montreal Cognitive Assessment test (MoCA) to complex neuropsychological batteries, with no consensus on interpretation thresholds or frequency of application [11]. In addition, most studies are cross-sectional, with small samples, and do not allow for the characterization of cognitive evolution in parallel with the progression of lung disease.

Despite the increasing awareness of cognitive dysfunction in other chronic respiratory diseases like COPD, evidence in IPF remains scattered and inconsistent. The lack of standardized assessment tools and the limited number of neuropsychological studies prevent the integration of cognitive evaluation into clinical practice. Therefore, understanding cognitive profiles in IPF could have direct effects on treatment adherence, rehabilitation participation, and patient autonomy.

This systematic review aimed to critically synthesize current evidence on cognitive impairment in IPF by integrating psychometric, physiological, and clinical data, while also assessing the certainty of the findings using standardized methodological appraisal tools like JBI and GRADE.

## 2. Materials and Methods

This paper is a systematic review conducted in accordance with the PRISMA 2020 (Preferred Reporting Items for Systematic Reviews and Meta-Analyses) guideline. The protocol was prospectively registered in the PROSPERO database under the code CRD420251041866. This study received ethical approval from the Ethics Committee of the “Dr. Victor Babeș” Clinical Hospital of Infectious Diseases and Pulmonology in Timișoara (Approval No. 12196, dated 17 December 2024). This study did not involve direct enrollment of human participants. All included studies had obtained informed consent from their respective participants.

We conducted a systematic literature search in four electronic databases: PubMed, Scopus, Web of Science, and The Cochrane Library, for the period from 1 January 2014, to 1 September 2025. The search strategy was designed to maximize the identification of relevant articles on cognitive impairment in patients diagnosed with idiopathic pulmonary fibrosis. To do this, terms were selected that combine the three main dimensions of interest: fibrosing interstitial lung disease, cognitive impairment, and methods used to assess cognitive function, with the use of Boolean operators “AND” and “OR” between concepts.

Keywords for IPF included the terms “idiopathic pulmonary fibrosis,” “IPF,” “fibrosing interstitial pneumonia,”. For cognitive impairment, terms such as “cognitive impairment”, “cognitive dysfunction”, “cognitive decline”, “mild cognitive impairment”, “memory”, “attention”, “executive function”, “processing speed”, “working memory” and “neurocognitive” were used. Regarding assessment instruments, the strategy included terms such as “MoCA”, “Montreal Cognitive Assessment”, “MMSE”, “Trail Making Test”, “TMT”, “Digit Span”, “Verbal Fluency”, “neuropsychological test” and “cognitive battery”. To cover terminological variability, truncations such as “cognit* impair*” and “neuropsycholog* test*” were used, and the query was limited to the occurrence of the terms in the title and abstract of the articles. In addition, bibliographies of included articles were manually reviewed to identify other relevant sources, and clinical trial registries, such as ClinicalTrials.gov and ISRCTN, were consulted to capture possible ongoing or unpublished work. To minimize publication bias and ensure comprehensive coverage, we also reviewed grey literature sources including clinical trial registries and conference proceedings.

### Study Selection and Data Extraction

A systematic search of PubMed, Scopus, Web of Science, and the Cochrane Library identified 134 articles. After removing 28 duplicates, 106 articles remained for screening based on title and abstract. Of these, 84 were excluded because they did not specifically address cognitive dysfunction or did not target patients with IPF.

**Figure 1:**
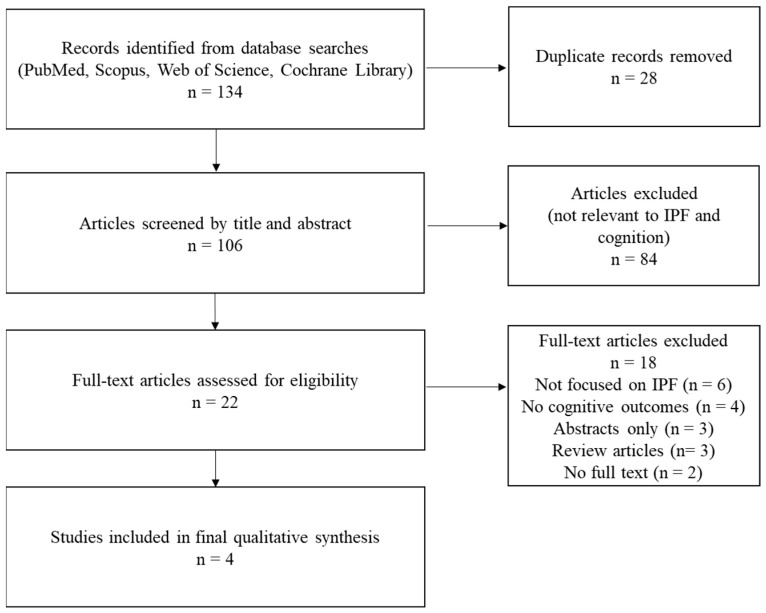
Flow diagram of the selection process.

A total of 18 articles were assessed in full text for eligibility. After removal of duplicates, all retrieved titles and abstracts were independently screened by two authors (A.C. and D.M.) based on predefined inclusion and exclusion criteria. Eligible full texts were then reviewed in detail. Any discrepancies between reviewers were resolved through discussion and consensus; when necessary, a third author (C.0.) was consulted to arbitrate. 18 articles were excluded for the following reasons: six did not address IPF, four did not report cognitive outcomes, three were available only in abstract form, three were reviews, and two did not have access to the full text. Ultimately, four studies met the inclusion criteria and were included in the qualitative synthesis (Figure 1).

The quality of the included studies was assessed using the tools developed by the Joanna Briggs Institute (JBI), specific to each type of study (cross-sectional, observational, descriptive). The scores were calculated as a proportion between the number of criteria met and the total number applicable to each [12]. Item-by-item results are summarized in a dedicated appraisal table, covering the four studies included in this review [6,7,9,11] (Supplementary Table S1).

The level of certainty of the evidence was assessed using the GRADE (Grading of Recommendations, Assessment, Development and Evaluation) [13]. This assessment was carried out independently by two authors, and any divergences were resolved by consensus. The justification for the JBI and GRADE scores is presented in Table 1 which details the instruments used, the cognitive domains investigated, and the clinical correlates reported. A summary of findings table was constructed to present the key outcomes, the certainty of evidence for each cognitive domain, and the main findings according to GRADE recommendations (Supplementary Table S2) reflecting the same body of evidence included in this review [6,7,9,11]. Due to the small number of included studies, a funnel plot to assess publication bias was not feasible.

## 3. Results

We reviewed four full-text articles that met the inclusion criteria (Figure 1). Of these, two were cross-sectional studies [7,9], one was a prospective descriptive study [6], and one was a case–control pilot study [14]. Together, these investigations provide the first evidence mapping the cognitive profile of patients with IPF. Despite methodological variability, all studies used validated neuropsychological assessments and included control groups. The most common tools were the Montreal Cognitive Assessment (MoCA), Trail Making Test (TMT A/B), Stroop, and the Hopkins or Rey Auditory Verbal Learning Tests. The overall methodological quality was moderate, with JBI scores of 6/8 across all studies. Certainty of evidence for each cognitive domain was graded using GRADE criteria, ranging from low to very low depending on the number of supporting studies and the internal consistency of findings (Supplementary Table S2).

### 3.1. Presence of Cognitive Impairment in IPF

Across all four studies, patients with idiopathic pulmonary fibrosis showed measurable declines in cognitive performance compared to healthy individuals. Tudorache et al. observed significantly lower global MoCA scores in IPF patients relative to controls (median 24 vs. 27 points, *p* = 0.003) [9]. Bors et al. similarly reported poorer outcomes in the severe IPF group compared to both mild IPF and matched controls, especially on tasks involving processing speed and divided attention, such as Trail Making Test B (mean completion time 135.9 s vs. 83.2 s in controls, *p* < 0.01) [6]. Giannouli et al. found that ILD patients, including those with IPF, performed notably worse than matched controls across multiple cognitive tasks despite similar demographic characteristics [7]. Additionally, Annaka et al. demonstrated that even patients with mild IPF scored lower than controls on measures of memory, attention, and executive functioning, including significantly impaired delayed recall on the RAVLT (*p* < 0.004) [14]. Overall, these findings consistently indicate that cognitive dysfunction is detectable in IPF throughout the disease spectrum.

### 3.2. Cognitive Domains Affected in IPF

The pattern of cognitive impairment varied across different domains, although several areas of dysfunction were consistently identified. Memory deficits were among the most commonly reported findings. Both Bors et al. and Annaka et al. described reductions in verbal learning and delayed recall [6,14], while Giannouli et al. identified impairments in both verbal and visual memory compared to controls [7]. Processing speed was also consistently affected, with longer completion times seen on Trail Making Test A/B and decreased performance on Stroop paradigms, especially in individuals with more advanced disease [6,7,14]. Executive dysfunction, presenting as difficulties in divided attention, interference control, and cognitive flexibility, was likewise observed across these studies. Additional findings included lower working memory performance [7], reduced visuospatial accuracy and constructional skills [9,14], and decreases in naming or fluency that were less consistently reported across studies [9,14]. Motor coordination issues, demonstrated by slower psychomotor and fine-motor performance, were mainly seen in severe IPF [6]. Overall, the strongest evidence pointed to impairments in memory, processing speed, and executive function, while visuospatial and language-related deficits were more variably reported. A summarized overview of the specific cognitive domains affected in IPF, along with their characteristic impairments and associated physiological correlates, is provided in Table 2.

### 3.3. Physiological and Clinical Correlations of Cognitive Impairment

Several physiological and clinical parameters showed significant links to cognitive performance in patients with idiopathic pulmonary fibrosis. In three studies, reduced diffusing capacity for carbon monoxide was the most consistent physiological factor. Lower DLCO levels correlated with poorer attention, slower processing speed, and decreased verbal fluency, suggesting that impaired gas-exchange efficiency may reflect or contribute to disruptions in neural networks that support higher-level cognitive functions [6,7,14].

Exercise capacity also played an important role. Giannouli et al. reported that shorter six-minute walk distance correlated with weaker memory performance and reduced psychomotor speed, indicating that diminished functional reserve may contribute to cognitive inefficiency through exercise-induced desaturation or impaired cardiovascular response [7].

Markers of oxygenation showed further links with cognitive outcomes. Lower end-walk oxygen saturation, more exertional desaturation, and higher post-exercise heart rate were associated with slower processing speed and less accuracy on recognition tasks in ILD and IPF groups [6,7]. Sleep-disordered breathing also seemed to affect cognitive outcomes. Tudorache et al. found that a higher apnea–hypopnea index and increased daytime sleepiness were independently linked to MoCA scores below 23, even after adjusting for demographic factors and lung function, suggesting that nocturnal hypoxia or sleep fragmentation may worsen cognitive vulnerability in IPF [9].

Clinical severity indicators further support these associations. Annaka et al. found that higher mMRC dyspnea scores and more advanced GAP stages were linked to worse performance in memory and executive functioning tasks [14]. Together, these findings suggest that reductions in diffusing capacity, limited exercise tolerance, abnormal oxygenation patterns, sleep-related breathing disturbances, and overall disease severity contribute to the cognitive phenotype seen in IPF.

## 4. Discussion

To our knowledge, this review is the first to systematically assess both methodological quality and certainty of evidence regarding cognitive impairment in idiopathic pulmonary fibrosis, using the JBI and GRADE frameworks to measure the strength and consistency of findings across cognitive domains. Across all included investigations, the collective evidence clearly supports the presence of cognitive impairment in IPF, establishing this phenomenon as a reproducible clinical feature rather than an incidental observation [6,7,9,14].

Across all available studies, patients with IPF performed worse on cognitive assessments than healthy controls or individuals with other chronic respiratory diseases. Bors et al. reported significant deficits in divided attention, psychomotor coordination, and verbal learning, especially in patients with severe IPF (DLCO < 30%) who needed oxygen therapy [6]. Giannouli et al. confirmed that lower DLCO and post-exercise desaturation were linked to poorer scores on tests of memory, attention, and processing speed [7]. Tudorache et al. found global cognitive decline, with more severe impairment in patients who also had obstructive sleep apnea [9]. Most recently, Annaka et al. showed that cognitive dysfunction can occur even in mild IPF, with lower performance on tasks involving verbal learning, delayed recall, and cognitive flexibility [14]. Together, these findings indicate that cognitive decline in IPF follows a progression, starting early and worsening as the disease advances and with comorbidities like OSA or frailty.

Across the four included original studies, cognitive impairment in IPF was consistently demonstrated, despite differences in samples and assessment tools. Bors et al. [6] and Giannouli et al. [7] used extended neuropsychological batteries and showed broad decrements spanning attention, processing speed, psychomotor coordination, memory, and naming/fluency, whereas Tudorache et al. [9] applied a global screener (MoCA) to examine the impact of comorbid OSA. Annaka et al. [14] added staged case–control data, showing that patients with more advanced physiological impairment (stage II–III) performed worse on processing-speed and interference tasks and on verbal learning, with significant group effects for Clock Drawing, RAVLT immediate recall, and Stroop/Reverse Stroop, and post hoc differences indicating poorer scores in stage II–III versus stage I or controls.

A common finding across studies was the relationship with disease severity. Lower diffusion capacity and oxygen therapy status identified patients with the slowest TMT and poorest pegboard performance; the effect was strongest when DLCO was below approximately 30 percent predicted or supplemental oxygen was needed [6]. Giannouli reported inverse correlations between cognitive scores and DLCO and FEV_1_/FVC, along with associations to post-exercise desaturation and heart rate, linking aerobic strain to executive and attentional inefficiency [7]. Annaka’s staging analysis aligned with this pattern: the stage II–III group, who also had higher mMRC scores and lower DLCO, performed worse on interference-based tasks and verbal learning compared to stage I and controls [14]. Complementing physiological severity, Tudorache showed that OSA comorbidity independently worsened overall cognitive status on the MoCA, even when resting spirometric values were acceptable, indicating an additional burden from nocturnal hypoxemia and sleep fragmentation [9].

While these associations are consistent, the underlying biological mechanisms remain insufficiently explored in the primary literature. Several mechanistic pathways may plausibly explain the vulnerability of specific cognitive domains identified in this review. Chronic hypoxemia and intermittent desaturation can impair cerebral oxygen delivery, leading to regional cerebral hypoperfusion, endothelial dysfunction, and oxidative stress, processes known to disproportionately affect the hippocampus and prefrontal cortex, the two regions most essential for verbal memory, working memory, processing speed, and executive control. Evidence from neurophysiology indicates that even moderate hypoxia can reduce hippocampal neurogenesis and impair long-term potentiation [15,16], while the prefrontal cortex is highly sensitive to fluctuations in oxygenation and inflammatory signaling [17]. These mechanisms align well with the domain profile observed in IPF. Intermittent desaturation, particularly with exertion or during sleep, may further generate cycles of oxidative injury, autonomic imbalance, and microvascular compromise, resembling the cognitive pattern described in OSA [18]. This may explain why patients with concomitant OSA, as reported by Tudorache et al. [9], present with more pronounced global deficits.

Systemic inflammation is another biologically plausible mechanism. IPF features chronic inflammatory activation, and circulating cytokines can cross the blood–brain barrier, activate microglia, and impair synaptic plasticity [19]. Similar lung–brain inflammatory pathways are observed in COPD and are increasingly seen as contributors to cognitive decline. Recent studies in COPD have identified the aging-related cytokine growth differentiation factor-15 (GDF-15) as a possible mediator linking physical inactivity, mitochondrial stress, and cognitive impairment [20]. Because COPD and IPF share features such as chronic hypoxemia, reduced physical activity, systemic inflammation, frailty, and progressive functional decline, it is biologically plausible that senescence-related factors like GDF-15, IL-6, TNF-α, and other inflammatory mediators may similarly influence the “lung–brain–muscle” axis in IPF. Although there are no specific biomarker studies on IPF yet, this is a promising area for future research.

Analyzing these converging mechanisms together provides a plausible neurobiological explanation for why memory and executive functions, which are highly sensitive to oxygen and inflammatory fluctuations, are consistently among the most affected in IPF.

Within this broader framework, reduced exercise capacity may not only indicate pulmonary limitation but also increase cerebral vulnerability through decreased neurotrophic support and diminished cerebrovascular reactivity, mechanisms described in chronic cardiorespiratory disease [21]. These interconnected pathways help explain why functions that require rapid information processing and flexible executive control, which depend on intact fronto-subcortical circuits, are disproportionately impaired in IPF.

In the context of these observations, cognitive impairment should not be viewed as an isolated phenomenon, but as a relevant component of the global clinical picture in IPF, with direct functional implications. Multidimensional assessment that integrates pulmonary parameters, exercise tests and neurocognitive status provides a more accurate picture of the impact of the disease on the quality of life and autonomy of patients [4,9,22,23].

This holistic approach is also supported by the ATS/ERS guidelines on the evaluation of patients with interstitial lung disease, which recommend screening for depression, anxiety, and cognitive function, along with functional testing [24,25].

Reduced attention, memory, and executive function may hinder adherence to oxygen therapy, recognition of symptom worsening, and active engagement in pulmonary rehabilitation [6,9,26,27]. Similar patterns seen in other chronic diseases, like heart failure and chronic kidney disease, highlight the prognostic significance of cognitive decline, which is linked to higher mortality, more hospitalizations, and poorer compliance with treatment plans [26,27,28].

Given these implications, systematic cognitive screening should be incorporated into multidisciplinary care, especially for moderate and severe IPF [9,23]. Brief, validated tools like the MoCA or Mini-Cog can efficiently detect subtle deficits, even when used by non-specialist staff, and can guide personalized therapeutic strategies and psychosocial support [29,30]. Early detection of cognitive impairment may also enable timely rehabilitation adjustments and enhance overall disease management [27,29,31].

Bridging the gaps: what comes next?

The first major limitation of the existing literature is the small number of studies that have systematically evaluated cognitive dysfunction in IPF, compared to other chronic respiratory pathologies such as COPD or OSA. In the PubMed database, there are over 10 times more studies related to “cognition + COPD” than to “cognition + IPF”, reflecting a clear gap in scientific interest in this area. Furthermore, most available studies are observational, with small samples and cross-sectional designs, which limits the possibility of establishing a causal relationship between the severity of lung disease and cognitive impairment.

Another important limitation is the lack of consensus on the optimal cognitive instruments for this population. Studies have used different tests (MoCA, MMSE, complex neuropsychological batteries), which reduces the comparability of results and prevents rigorous meta-analyses. For example, the MMSE is recognized as less sensitive to impairment of executive functions and attention—domains frequently altered in chronic hypoxia—while the MoCA is more appropriate but less widely used. Also, possible confounding factors—such as depression, anxiety, pharmacological treatments, or sleep disorders—are not controlled or consistently reported in current studies. Psychological distress, including symptoms of depression and anxiety, is frequently reported in patients with idiopathic pulmonary fibrosis and can negatively influence cognitive test performance, potentially masking or exaggerating true neurocognitive impairment [32]. Less explored are the brain imaging changes associated with IPF, although some studies have begun to investigate the relationships between hypoxia, vascular microlesions, and cortical atrophy using functional MRI or DTI [33,34].

The present review adds novelty by being, to our knowledge, the first to quantitatively evaluate both methodological quality and certainty of evidence across cognitive domains using the JBI and GRADE frameworks. This approach allows a clearer understanding of the relative robustness of available data and identifies domains where the certainty remains low or very low, especially for visuospatial and language functions.

In terms of future directions, the following are needed:

- Longitudinal studies to track cognitive evolution over time and identify early predictors of decline.
- Targeted interventions, such as cognitive exercises combined with respiratory rehabilitation, to assess the impact on cognitive function and quality of life.
- Integrating inflammatory or imaging biomarkers to better understand the neurobiological substrate of cognitive dysfunction in IPF.
- Evaluating the impact of antifibrotic therapy on cognition, considering potential indirect effects through amelioration of hypoxia and reduction in exacerbations.

## 5. Conclusions

Idiopathic pulmonary fibrosis consistently linked to measurable cognitive impairment, most often affecting working memory, verbal learning, processing speed, and executive function. These deficits are seen throughout the disease spectrum, including mild IPF, and are clinically significant because they may impact treatment adherence, daily activities, and participation in rehabilitation.

At the same time, the certainty of the current evidence remains low to very low according to the GRADE assessment, primarily due to the small number of available studies, modest sample sizes, and heterogeneity in cognitive testing methods. This limitation highlights an urgent need for high-quality, prospective, and mechanistic studies to accurately determine the prevalence, trajectory, and clinical implications of cognitive dysfunction in IPF.

Despite these limitations, short cognitive screening tools like the MoCA may assist clinicians in identifying early deficits and integrating cognitive factors into personalized management and rehabilitation plans for patients with IPF.

## Figures and Tables

**Table 1:** Characteristics of the included studies on cognitive function in IPF.

Authors	Design	IPF Sample Size	Age Range (Years)	Cognitive Tests	Main Cognitive Domains Affected	Associated Clinical Factors	JBI	Grade
Bors et al. [6]	Prospective descriptive study	46 IPF (moderate n = 34 Severe n = 12) Control (n = 15)	Moderate IPF = 63.2 ± 9.6; Severe = 69.3 ± 9.4; Controls = 66.0 ± 10.8	Trail Making Test A/B, Stroop Color-Word (1–3), Hopkins Verbal Learning Tes–Delayed Recall, Boston Naming Test, Grooved Pegboard (a/b)	Divided attention (TMT-B), processing speed (TMT-A, Stroop), psychomotor coordination (Pegboard), verbal memory (HVLT-DR)	Severe IPF (DLCO ≈ 20% pred., all on home O_2_) showed slower TMT-B (135 s vs. 86/83; *p* < 0.01) and Stroop 3 (22 vs. 30 vs. 38; *p* < 0.01), poorer Pegboard performance (>120 s vs. ≈80 s; *p* < 0.01), and higher BDI-II scores; greater disease severity correlated with lower HRQoL (SF-36)	6/8	Low–very low
Tudorache et al. [9]	Cross-sectional study	IPF (n = 23) Control (n = 17)	IPF = 67.6 ± 8.7; Controls = 60.6 ± 7.3	MoCA (global + subdomains)	Delayed recall, language, naming, and visuospatial abilities significantly impaired in IPF (*p* < 0.05 each)	Higher OSA severity (AHI 33 vs. 12, *p* = 0.018) and greater daytime sleepiness (Epworth 6.5 vs. 3, *p* = 0.013) in IPF with MoCA < 23; no associations with age or lung function parameters	6/8	Low–very low
Giannouli et al. [7]	Cross-sectional study	51 ILD patients (including 15 IPF) Controls (n = 49)	ILD = 61.2 ± 12.3; Controls = 63.7 ± 12.5	Rey–Osterrieth Complex Figure Test, Word List Learning Test, Trail Making Test A/B, Digit Span (forward/backward), Verbal Fluency (semantic and phonological), Ruff 2 & 7 Selective Attention Test, Geriatric Depression Scale	Verbal and visual memory, delayed recall, and recognition scores significantly lower in ILD; psychomotor speed reduced (TMT A/B); attention and executive accuracy (Ruff 2 & 7) relatively preserved	Poorer cognition associated with lower DLCO% (β = 0.58, *p* = 0.011), shorter 6MWD (β = −0.58, *p* = 0.047), higher HR post-exercise (β = −0.57, *p* = 0.015), and lower end-walk SpO_2_ (β = −0.65, *p* = 0.005)	6/8	Low–very low
Annaka et al. [11]	Case–control pilot study	20 IPF (mild n = 10, mod-severe n = 10) Controls (n = 16)	Controls = 69.0 ± 1.4; Stage I = 66.6 ± 1.4; Stage II–III = 67.7 ± 1.5	MMSE, Animal Naming, Clock Drawing, Digit Span (forward/backward), RAVLT (recall/delayed), Trail Making Test A/B, Reverse Stroop, Stroop	Verbal learning and delayed recall (RAVLT, *p* = 0.045–0.047), processing speed and cognitive flexibility (Stroop *p* = 0.009; Reverse Stroop *p* = 0.030)	Lower MMSE and Stroop scores in stage II–III vs. stage I (MD = 1.15; *p* = 0.013); higher mMRC (*p* < 0.001) and lower DLCO (46.5% vs. 67.1%, *p* < 0.001) associated with poorer cognition	6/8	Low–very low

Abbreviations: AHI—Apnea–Hypopnea Index; BDI-II—Beck Depression Inventory-II; DLCO—Diffusing capacity of the lung for carbon monoxide; HR—Heart rate; HRQoL—Health-related quality of life; HVLT-DR—Hopkins Verbal Learning Test—Delayed Recall; ILD—Interstitial lung disease; IPF—Idiopathic pulmonary fibrosis; mMRC—Modified Medical Research Council Dyspnea Scale; MoCA—Montreal Cognitive Assessment; MMSE—Mini-Mental State Examination; OSA—Obstructive sleep apnea; RAVLT—Rey Auditory Verbal Learning Test; 6MWD—Six-minute walk distance; SF-36—Short Form-36 Health Survey; SpO2—Peripheral oxygen saturation; TMT-A/B—Trail Making Test A and B.

**Table 2:** Summary of cognitive domains affected in idiopathic pulmonary fibrosis.

Cognitive Domain	Main Findings Across Studies	Typical Clinical Correlates	Consistency
Memory (verbal/visual)	Reduced immediate and delayed recall; impaired learning and recognition.	↓ DLCO %, shorter 6-MWD, ↓ end-walk SpO_2_, ↑ post-exercise HR.	High [6,7,11]
Processing speed	Slower TMT A/B completion times.	Disease severity, oxygen-desaturation indices.	Moderate [6,7,11]
Working memory	Lower Digit Span scores.	Correlated with DLCO % and 6-MWT parameters.	Moderate [7]
Executive function/attention	Divided attention and interference deficits.	Advanced disease; hypoxemia.	Moderate [6,11]
Visuospatial/constructional	Impaired figure-copy accuracy.	↓ DLCO %, ↓ SpO_2_.	Moderate [6,9]
Language	Mild naming/fluency reduction.	Higher OSA severity and daytime sleepiness.	Low [9]
Motor coordination	Slower fine-motor performance.	Severe IPF; all on home O_2_.	Low [6]
Global cognition	Mild overall reduction.	Linked with OSA severity only.	Low [9]

Consistency across studies was graded qualitatively based on the replication of domain-specific impairments: High—reported in ≥3 studies with convergent findings; Moderate—reported in 2 studies or with partial overlap; Low—observed in a single study without replication. Abbreviations: DLCO—diffusing capacity of the lung for carbon monoxide; HR—heart rate; IPF—idiopathic pulmonary fibrosis; OSA—obstructive sleep apnea; SpO_2_—peripheral oxygen saturation; TMT—Trail Making Test.

## Data Availability

No new data were created or analyzed in this study.

## Supplementary Materials

The following supporting information can be downloaded at: https://www.mdpi.com/article/10.3390/medsci13040288/s1, Supplementary Table S1: Item-by-item JBI grading for the included studies. Supplementary Table S2: Certainty of evidence for cognitive domains in the included studies.

## Abbreviations

The following abbreviations are used in this manuscript:

Abbreviation	Meaning
6MWD	Six-minute walk distance
AHI	Apnea–Hypopnea Index
BDI-II	Beck Depression Inventory-II
COPD	Chronic Obstructive Pulmonary Disease
DLCO	Diffusing capacity of the lung for carbon monoxide
FEV_1_	Forced expiratory volume in one second
FVC	Forced vital capacity
GRADE	Grading of Recommendations, Assessment, Development and Evaluation
HR	Heart rate
HRQoL	Health-related quality of life
HVLT-DR	Hopkins Verbal Learning Test—Delayed Recall
ILD	Interstitial lung disease
IPF	Idiopathic pulmonary fibrosis
JBI	Joanna Briggs Institute
mMRC	Modified Medical Research Council Dyspnea Scale
MoCA	Montreal Cognitive Assessment
MMSE	Mini-Mental State Examination
OSA	Obstructive sleep apnea
PRISMA	Preferred Reporting Items for Systematic Reviews and Meta-Analyses
PROSPERO	International Prospective Register of Systematic Reviews
RAVLT	Rey Auditory Verbal Learning Test
SF-36	Short Form-36 Health Survey
SpO_2_	Peripheral oxygen saturation
TMT-A/B	Trail Making Test A and B
