# Supplementary material for: Neurocognitive Impairment in Idiopathic Pulmonary Fibrosis: A Systematic Review of Current Evidence
Source: Med Sci (Basel). 2025 Nov 27;13(4):288. doi: 10.3390/medsci13040288 (PMC12735246; doi:10.3390/medsci13040288)
Supplement: Supplementary file 1 [file medsci-13-00288-s001.zip › Supplementary Table S1.pdf]

Supplementary Table S1. Item-by-item JBI grading for the included studies

**JBI Item**	**Bors (2015)**	**Tudorache (2019)**	**Giannouli (2021)**	**Annaka (2025)**
1. Were the criteria for inclusion in the sample clearly defined?	Yes	Yes	Yes	Yes
2. Were the study subjects and the setting described in detail?	Yes	Yes	Yes	Yes
3. Was the exposure measured in a valid and reliable way?	Yes	Yes	Yes	Yes
4. Were objective, standard criteria used for measurement of the condition?	Yes	Yes	Yes	Yes
5. Were confounding factors identified?	Partial	Partial	Partial	Partial
6. Were strategies to deal with confounding factors stated?	No	No	Limited	Limited
7. Were the outcomes measured in a valid and reliable way?	Yes	Yes	Yes	Yes
8. Was appropriate statistical analysis used?	Yes	Yes	Yes	Yes
**Total**	6/8	6/8	6/8	6/8
