# Supplementary material for: Neurocognitive Impairment in Idiopathic Pulmonary Fibrosis: A Systematic Review of Current Evidence
Source: Med Sci (Basel). 2025 Nov 27;13(4):288. doi: 10.3390/medsci13040288 (PMC12735246; doi:10.3390/medsci13040288)
Supplement: Supplementary file 1 [file medsci-13-00288-s001.zip › Supplementary Table S2.pdf]

Supplementary Table S2. Certainty of evidence for cognitive domains in the included studies

**Outcome (Domain)**	**No. of studies**	**Study design**	**Risk of bias**	**Inconsistency**	**Indirectness**	**Imprecision**	**Publication bias**	**Other considerations**	**Final certainty**	**Effect / Notes**
**Working memory**	2	Observational	Serious (–1)	No serious inconsistency	No serious indirectness	Serious (–1)	Not detected	None	⋆⋆◯◯ Low	Lower Digit Span scores in ILD (Giannouli 2022); working-memory trend in verbal tasks (Bors 2015) but not systematically tested in IPF.
**Verbal memory**	4	Observational	Serious (–1)	No serious inconsistency	No serious indirectness	Serious (–1)	Not detected	None	⋆⋆⋆◯ Moderate	Consistent impairment in immediate and delayed

										recall (Bors 2015; Giannouli 2022; Annaka 2025); supported by MoCA delayed-recall subdomain in IPF (Tudorache 2019).
**Visual memory**	2	Observational	Serious (–1)	No serious inconsistency	No serious indirectness	Serious (–1)	Not detected	None	⋆⋆◯◯ Low	Rey–Osterrieth figure recall and recognition lower in ILD (Giannouli 2022); no specific visual memory tests in

										other studies.
**Attention / executive function**	3	Observational	Serious (–1)	Moderate inconsistency (–1)	No serious indirectness	Serious (–1)	Not detected	None	⋆◯◯◯ Very low	Deficits in divided attention and interference control (Stroop, TMT-B) reported in Bors 2015 and Annaka 2025; not replicated in Tudorache or Giannouli.
**Processing speed**	3	Observational	Serious (–1)	No serious inconsistency	No serious indirectness	Serious (–1)	Not detected	None	⋆⋆◯◯ Low	Slower completion on TMT A/B and Stroop tasks (Bors 2015; Annaka

										2025); partly supported by Giannouli 2022.
**Language**	2	Observational	Serious (–1)	No serious inconsistency	No serious indirectness	Serious (–1)	Not detected	None	⋆⋆◯◯ Low	Reduced naming and verbal fluency in IPF (Tudorache 2019; Giannouli 2022); small effect sizes and task dependence.
**Visuospatial / constructional**	2	Observational	Serious (–1)	No serious inconsistency	No serious indirectness	Serious (–1)	Not detected	None	⋆⋆◯◯ Low	Impaired Rey–Osterrieth figure-copy accuracy (Giannouli 2022);

										MoCA visuospatial deficits confirmed in IPF (Tudorache 2019).
